# Current status and future perspectives of the diagnostic of plant bacterial pathogens

**DOI:** 10.3389/fpls.2025.1547974

**Published:** 2025-02-28

**Authors:** Xu Wang, Shuiying Liang, Qinhua Gan, Bo Cai, Caixia Liu

**Affiliations:** ^1^ School of Tropical Agriculture and Forestry, Hainan University, Hainan, China; ^2^ Post-Entry Quarantine Station for Tropical Plant, Haikou Customs District, Hainan, China; ^3^ Hainan Provincial Key Laboratory of Tropical Hydrobiotechnology, Hainan University, Haikou, China; ^4^ Technology Center of Qingdao Customs District, Qingdao, China

**Keywords:** plant pathogens, morphology-based diagnostic, immunology-based methods, micro-Raman spectroscopy, non-invasive discrimination, single-cell detection

## Abstract

Diagnostic of plant bacterial pathogens underwent a leapfrog development from culture-based strategies to culture-free detection. Conventional diagnostics, such antibody- and PCR-based methods, are sensitive to identify pre-enriched pathogens in naturally infected crops at the late stage. However, they suffer from shortcomings relating to rapidity, signal strength, and a significant reduction in sensitivity in real plant extract. Progress has been made to address these challenges through development of labelled and non-labelled optical spectroscopy. Specifically, the micro-Raman spectroscopy enables fast, label-free, and non-invasive discrimination of viable but non-culturable pathogens at a single-cell level. A comprehensive spectroscopic database is always a prerequisite for identification, yet these spectroscopy-based methods are insufficient to detect previously unknown plant pathogens. The advance of single-cell sequencing and synthetic biology is beginning to address these crucial problems and is being used in related practical applications. Success will continue to be found at the interfaces between disciplines.

## Crop pathogens and global food security

**Figure 1:**
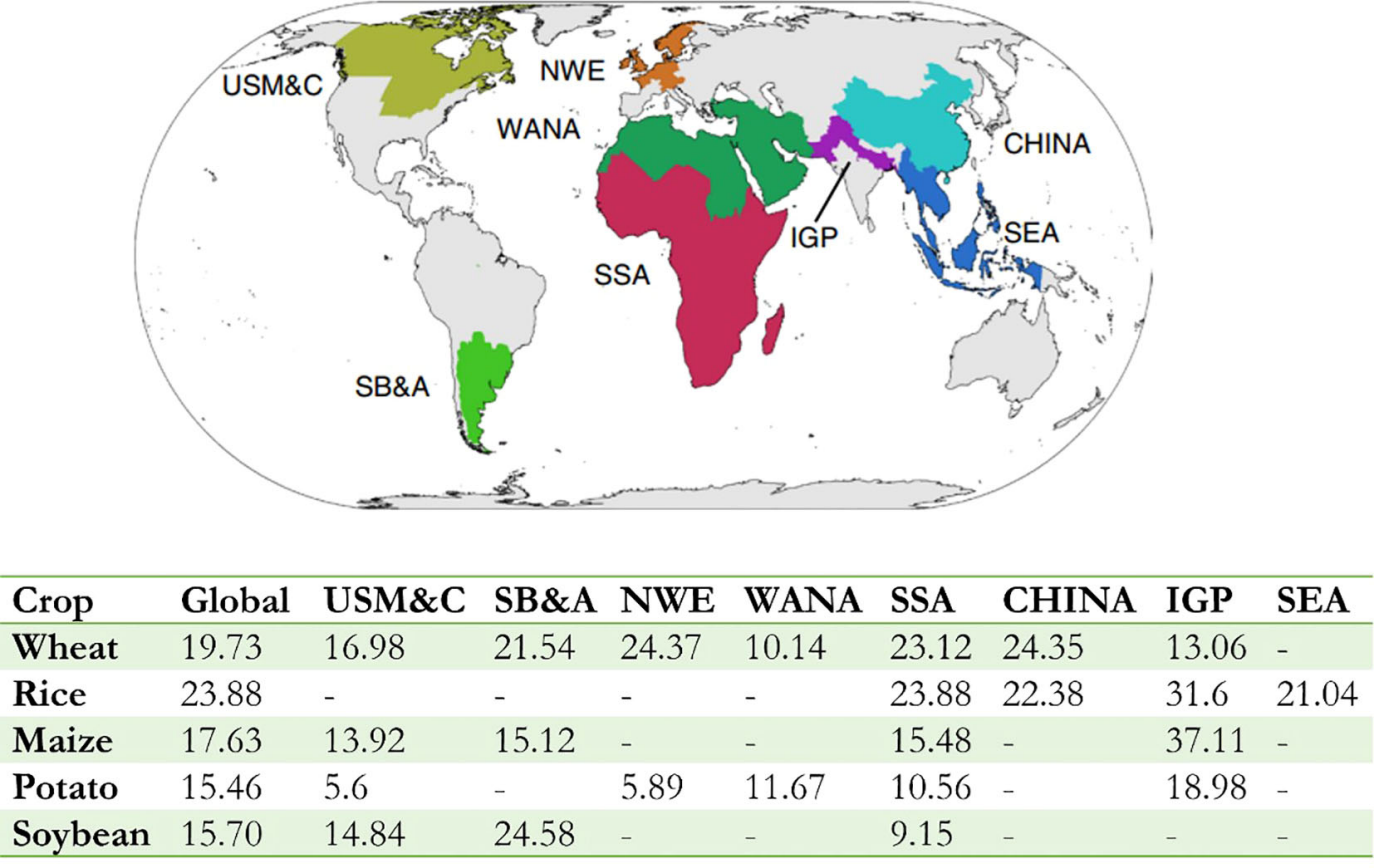
Global variations in crop losses and production. The global map shows the location of the eight food security hotspots where there were sufficient survey responses to estimate the loss (Hijmans et al., 2018). The bottom table shows losses for wheat, rice, maize, potato, and soybean globally or specifically to each food security hotspot. It can be see that crop losses are lower in hotspots generating food surpluses (USM&C, SB&A and NWE, except for soybean) and higher in hotspots located in food insecure regions (SSA and IGP) frequently. Food security hotspot: USM&C, US Midwest and Canada; SB&A, South Brazil, Paraguay, Uruguay and Argentina; NWE, Northwest Europe; WANA, West Asia and North Africa; SSA, Sub-Saharan Africa; CHINA, Mainland China; IGP, Indo-Gangetic Plain; SEA, Southeast Asia. [Adapted from previous study (Savary et al., 2019)].

The world population is expected to rise from 7.2 billion to 9.7 billion by 2050 and 11 billion at the end of the century (Population Division of the Department of Economic and Social Affairs of the United Nations Secretaria, 2022). Global agricultural production must increase by 70% and 106% to satisfy a growing demand for food. However, the epidemic of crop pests and pathogens causes substantial economic losses and reduce food security at household, national, and global levels. Despite the difficulty in quantitative, standardized assessment of crop losses due to disease across crops, it was estimated to average 21.5% in wheat, 30.0% in rice, 22.6% in maize, 17.2% in potato, and 21.4% in soybean yield losses at a global level (Savary et al., 2019) while these crops account for half of the global human calorie intake (Food and Agriculture Organization of the United Nations, 2024). The highest losses are associated with food-deficit regions with fast-growing populations (e.g., Indo-Gangetic Plain and Sub-Saharan Africa), and frequently with emerging or re-emerging diseases (**Figure 1**) (Savary et al., 2019). The situation is aggravated by global trade which intensifies the spread and distribution of quarantine pathogens (Elad and Pertot, 2014), many of which can spread or reemerge after having been under control (Bhattacharya, 2017). Secondary yield losses caused by the negative impacts of pests and diseases in the previous year was even worse (Cerda et al., 2017). Approximately 110 billion kilograms of food loss, 1.5 billion kilograms of cotton, 2.3 billion kilograms of oil, 50 billion kilograms of vegetables, and 6 billion kilograms of fruits could be saved by pest control each year, which is equivalent to an increase of 12% to 18% of the planting area. However, many countries, particularly low-income countries, are incompetent in monitoring and preventing disease spread. Therefore, to improve responses to unexpected crop disease spread and minimize the risk to food supplies, a global surveillance system for crop diseases is critical, which will extend and adapt established biosecurity practices and networking facilities (Carvajal-Yepes et al., 2019).

The occurrence and prevalence of plant diseases vary from season to season, depending on the presence of the pathogen, environmental conditions, and the crops and varieties grown. Survival improves when pathogen is detected early. However, majority of plant diseases are at an advanced stage when diagnosed. Early detection of pathogen allows early intervention to try to slow or prevent disease development and lethality. To achieve early detection of all pathogen, numerous of methods have been developed. In this article, we review the development of the plant pathogen detection technology and briefly summarize the challenges and the future directions. We see how our existing knowledges about the pathogen biology and technology development promote the sensitivity, accuracy, and throughput of detection methods for plant pathogens. Interdisciplinary collaboration is key in transforming progress in technology and biology of plant pathogens to improve early detection and plant survival.

## Morphology-based techniques

Conventional methods for the detection and identification of plant pathogens mainly rely on recognizing specific morphological features, which involves a time-consuming and labor-intensive process for pathogen cultivation (Camargo and Smith, 2009; Li et al., 2012; Li et al., 2014b; Li et al., 2014a) (**Table 1**). The period of time from the infection of pathogens to the observation of the syndromes of suspected infectious etiology is variable ranging from days for fast-growing bacteria to weeks for slower growing species (Qinhua et al., 2011). On the other hand, individual pathogens may cause a variety of plant diseases with a wide range of pathological phenotypes which are hard to standardize and have high false positive rates. Occasionally certain pathogens cause subtle symptoms in the tolerant crop cultivar but can have devastating effects in a susceptible one. The situation is among others further aggravated by the morphology similarity that may be caused by different pathogens. The diagnosis of pathogens from the plant host can be complicated by the fact that plants may be infected by multiple pathogens simultaneously or individually in nature or in a laboratory setting (Camargo and Smith, 2009). Considering the largely identical morphological symptoms, the identification of different pathogens from the same plant host can be difficult. Moreover, the morphology-based detection methods rely heavily upon cultivation methods. Although cultivation methods have been improved considerably over the past several decades with advances in the scope and diversity of media components, it still involves a time-consuming process of culture enrichment. Moreover, certain plant pathogens do not readily grow in a laboratory environment. We would thus observe strong bias toward bacteria that are amenable to cultivation. Therefore, morphology-based techniques have very low sensitivity and accuracy and are not suitable for detection of unculturable pathogens that are also not easily viewed by light microscopes such as viruses, viroids, and phytoplasmas.

**Table 1:** Summary of various techniques used for plant bacterial pathogen detection.

Approaches	Advantages	Limitations	Detection limit (CFU/mL)	Instrumentation	Cost
**Morphology-based (** Camargo and Smith, 2009; Qinhua et al., 2011; Li et al., 2012; Li et al., 2014b; Li et al., 2014a)	Low cost	Experience-dependent, time-consuming, very low sensitivity and accuracy	10^7^-10^8^	Simple	Low
**Immunology-based (** Holgate et al., 1983; Van Laere et al., 1985; Wyatt et al., 1989; Mazarei and Kerr, 1990; Franken et al., 1992; Gallo et al., 2000; Anil et al., 2008; Yao et al., 2009; Braun-Kiewnick et al., 2011; Lattanzio et al., 2012; Charlermroj et al., 2013; Gan et al., 2013)	More specific, sensitive, reproducible, and reliable	Antigen-dependent, time-consuming sample preparation, and incapable of detecting unknown pathogens	10^5^-10^6^	Simple	Low
**Nucleic-acid-based (** Bereswill et al., 1992; De Boer, 1995; Mcmanus and Jones, 1995; Pulawska, 1997; Hélias et al., 1998; Manulis et al., 1998; Zaccardelli et al., 2003; Kong et al., 2004; Zaccardelli et al., 2005; Kumagai and Fabritius, 2008; Kumagai and Fabritius, 2008; Ning et al., 2008; Shenge et al., 2008; Haan E.G.d and Bovenkamp G.W.v.d, 2009; Pang et al., 2009; Park et al., 2009; Pirc et al., 2009; Shao et al., 2009; Qinhua et al., 2011; Shao et al., 2011; Araujo et al., 2012; Gašić et al., 2012; Gehring and Geider, 2012; Li et al., 2012; Štajner et al., 2013; Feng et al., 2013; Gan et al., 2013; Liu et al., 2013; Gan et al., 2014a; Gan et al., 2014b; Gan et al., 2014c; Li et al., 2014b; Li et al., 2014a; Luqi et al., 2014; Feng et al., 2015; Silva et al., 2015; Wang et al., 2015a; Wang et al., 2015b; Fan et al., 2016; Ilicic et al., 2016; Meng et al., 2016; Zhao et al., 2016; Yang et al., 2025)	Rapid, specific, and highly sensitive	Instrument-dependent and low sensitivity for pathogens in the presence of plant extract	10^3^-10^4^	Simple	Low
**Nanotechnology-based (** Meng et al., 2005; Yao et al., 2009; Sharon et al., 2010; Etefagh et al., 2013)	High sensitivity	High cost	1-10	Complicate	High
**Fluorescent hybridization-based (** Franken et al., 1992; Anil et al., 2008; Yao et al., 2009; Braun-Kiewnick et al., 2011; Lattanzio et al., 2012)	High sensitivity, rapid, straightforward, and capable to detect viable but non-culturable microbes	Label/device-dependent and difficult to find proper labels and determine proper labelling conditions	10^3^	Complicate	High
**Noninvasive optical spectroscopy techniques (** D’Haese and Nelis, 2002; Cools et al., 2005; Guicheteau et al., 2008; Jarvis and Goodacre, 2008; Blackie et al., 2009; Vanhee et al., 2009; Cam et al., 2010; Kang et al., 2010; Tebaldi et al., 2010; Kloss et al., 2013; Li AY. et al., 2014; Kloß et al., 2015; Kubryk et al., 2015; Yüksel et al., 2015; Lau et al., 2016; Wang et al., 2016; Gan et al., 2017; Li et al., 2017)	At single-bacterium level and without pre-enrichment	Signal noise and incapable of detecting unknown pathogens	1-10	Simple	High
**Single-cell sequencing (** Gan et al., 2017; Li et al., 2019; Marcolungo et al., 2022)	Detect at single-cell level rapidly without enrichment	Difficult to obtain single cells	1	Simple	High
**Synthetic biosensor (** Frechon et al., 1998; Llop et al., 2000; Taylor et al., 2001; Puławska and Sobiczewski, 2005; Mirik and Aysan, 2009; Abd-Elsalam et al., 2011; Kositcharoenkul et al., 2011; Kang et al., 2014; Pardee et al., 2014; Streets et al., 2014; Umesha et al., 2015; Mukherjee and Schroeder, 2016; Keller and Pantel, 2019; Garg et al., 2022; Attaluri and Dharavath, 2023; Cubero et al., 2024; Cao et al., 2025)	Low-cost, practical, and simple	Incapable of detecting unknown pathogens	1-10	Simple	Low

## Immunology-based methods

The sensitivity for cultivation-amenable microorganisms have been dramatically improved with the development of immunological detection relying mainly on the specific binding of an antibody to an antigen. The immunogold staining (IGS) was begin to be applied in 1985 to identify the plant pathogenic bacterium *Erwinia amylovora* (Van Laere et al., 1985). It requires less primary antiserum and shows the advantage that the preparations can be conserved permanently and unchanged. An “indirect” immunohistological technique was developed by employing immunoglobulin adsorbed to colloidal gold as the secondary antiserum. The technique, the immunogold-silver staining (IGSS), is of much enhanced sensitivity (up to 200-fold) as compared with IGS (Holgate et al., 1983). To further improve the sensitivity, an enzyme‐linked immunosorbent assay (ELISA) was developed where the detection limit of *Pseudomonas syringae* pv. *phaseolicola*, the agent of halo blight disease of beans, was as high as 2 × 10^4^ cells ml^−1^ (Wyatt et al., 1989; Gan et al., 2013). Indirect ELISA based on polyclonal antibodies was also developed to distinguish pathovars of *Pseudomonas syringae* from pea (Mazarei and Kerr, 1990). The suitability of the antigen-antibody complex depends mainly on the antibodies’ specificity. In order to ensure the reliable detection of pathogens, a variety of antibodies have been employed in different assay types. Most polyclonal antibodies, derived from either rabbit or goat serum, contain a collection of antibodies with different cellular origins and, therefore, somewhat different specificities. Monoclonal antibodies are often more useful than polyclonal ones for specific detection of a molecule, since they provide an indefinite supply of a single antibody. Lots of monoclonal antibodies have been commercialized. Specific polyclonal and monoclonal antibodies were developed for the identification of *Xanthomonas campestris* pv. *Campestris* and *Pseudomonas syringae* pv. *pisi*, with an enzyme immunoassay (EIA), immunofluorescence microscopy (IF), or a dot-blot immunoassay (DBI) (Franken et al., 1992).

With the development of monoclonal antibodies, immunological detection of microbial contamination has become more specific, sensitive, reproducible, and reliable (**Table 1**). When detecting *Pseudomonas syringae*, the immuno-assay method (competitive ELISA) is approximately 100 times more sensitive than the High Performance Liquid Chromatography (HPLC) method and requires no previous extraction (Gallo et al., 2000). On the other hand, the influence of sample preparation and antibody reactions should be thoroughly examined and understood first, as the detection sensitivity is often altered with the variability of these parameters. For example, comparison of dilution-plating by two different sample extraction methods revealed that, for samples of *Pseudomonas syringae* pv. *Pisi* in pea seed, methods involving longer time of soak in water for pea seeds were more sensitive (Gallo et al., 2000).

With the development of the immunological methods, reliable quantitative assessments detect plant pathogens were allowed to be managed in a high-throughput manner. Immune-dipstick and immune-lateral flow assays were developed and used in laboratories and then for field application. ELISA, immunofluorescence staining test (IFST), seed immunoblot binding assay (SIBA), dyed latex bead agglutination test, lateral-flow immunoassay, fluorescent silica nanoparticles (FSNP), and strip immunoassay have been successfully used to detect plant pathogens such as *Xanthomonas axonopodis* pv. *vesicatoria* (causing bacterial spot disease in tomatoes and peppers) (Yao et al., 2009) and *E. amylovora* (Braun-Kiewnick et al., 2011), with the detection limit equivalent to the typical of pathogen concentrations in symptomatic plant material (Anil et al., 2008).

To facilitate simultaneous detection of multiple pathogens, a multiplex dipstick immunoassay was developed by determining different major *Fusarium* toxins in wheat, oats, and maize (Lattanzio et al., 2012). An antibody for each plant pathogen was linked on a fluorescence-coded magnetic microsphere set which was used to capture corresponding pathogens (Lattanzio et al., 2012). The method was optimized by employing microsphere immunoassays where four important plant pathogens (i.e., fruit blotch bacterium *Acidovorax avenae* subsp. *citrulli*, chilli vein-banding mottle virus, watermelon silver mottle virus, and melon yellow spot virus) were simultaneously detected with substantially higher sensitivity and within much shorter assay time than ELISA (Charlermroj et al., 2013). The system was also shown to be capable of detecting pathogens in naturally infected plant samples. However, antibody-based detection is antigen-dependent and is still lacking the ability to detect plant pathogen in “real-time” for early infection detection and the timeline of disease progression.

## Nucleic-acid-based approaches

Nucleic-acid-based approaches are rapid, specific, and highly sensitive and are among the most useful and efficient methods available for phytopathogen detection (Li et al., 2012; Gan et al., 2014a; Li et al., 2014b; Li et al., 2014a; Wang et al., 2015a) (**Table 1**). Polymerase chain reaction (PCR) has been demonstrated to be rapid and accurate to detect wide array of pathogens [e.g., *E. amylovora* (Bereswill et al., 1992), *Agrobacterium tumefaciens* (Pulawska, 1997), *Pseudomonas marginalis* (Qinhua et al., 2011), *Xanthomonas campestris* pv. *Vesicatoria* (Gan et al., 2014b) and different *Pseudomonas syringae* pathovars (Zaccardelli et al., 2003; Kong et al., 2004; Zaccardelli et al., 2005)], where detection limits ranging from 250 to 500 CFU mL^-1^ were obtained (De Boer, 1995). Since the invention of PCR, numerous of derivatives have been developed to improve the simplicity, sensitivity, accuracy of pathogen detection. Molecular methods, such as restriction enzyme digestion, have been employed to simplify the pathogen detection where PCR-RFLP (Restriction Fragment Length Polymorphism) has been applied in detection of a numerous of phytopathogens from plant tissues, soil, and water extracts (Hélias et al., 1998; Shenge et al., 2008). Sample preparation, such as genomic DNA extraction, is time-consuming while automated DNA-extraction methods have been developed. Combined with the Real-time quantitative PCR (qPCR) assays, the detection method was able to discriminate *E. amylovora* (isolated from blighted woody plant material) from the other *Erwinia* strains (isolated from Hokkaido or necrotic pear blossoms) with a limit as low as 10^3^ cells mL^−l^ (i.e., four cells per reaction) (Pirc et al., 2009; Shao et al., 2009; Gan et al., 2013; Liu et al., 2013; Gan et al., 2014c). qPCR also showed high sensitivity when was used to differentiated *Verticillium wilt* on susceptible and resistant hop cultivars (*Humulus lupulus* L.) (Štajner et al., 2013), *Saccharothrix yanglingensis* Hhs.015 (a major apple Valsa canker pathogen) (Fan et al., 2016), and *Pseudomonas syringae* pv. *lachrymans* (in cucumber seeds) (Meng et al., 2016). TaqMan-PCR (Shao et al., 2011; Wang et al., 2015b) and loop-mediated isothermal amplification (LAMP) (Pang et al., 2009; Feng et al., 2013; Feng et al., 2015) have been developed to improve the sensitivity while a multiplex detection system could detect as low as 0.04 pg genomic DNA of fungal and oomycete pathogens of solanaceous crops (Ning et al., 2008). The sensitivity is able to be further enhanced by 1000-fold by employing nested PCR [is sufficient for single-cell detection in pure culture (Mcmanus and Jones, 1995)] or semi-selective medium prior to PCR [Bio-PCR, with a detection limit of five cells from pure culture (Manulis et al., 1998; Kumagai and Fabritius, 2008; Silva et al., 2015)]. Most recently, droplet digital PCR (ddPCR) began to be used for plant pathogen detection where ddPCR showed a significantly higher degree of sensitivity compared to the qPCR assay and the influence of PCR inhibitors can be reduced considerably in the ddPCR assay (Zhao et al., 2016). However, it is too instrument-dependent (Zhao et al., 2016) and technique-demanding.

PCR-based technology has been developed to detect multiple plant pathogens simultaneously. Primer design is very important, for it’s a component of the development of all nucleic acid-based methods. For example, repetitive sequences were used to distinguish different genetic profiles within *P. syringae* pathovars *P. s.* pvs. *syringae*, *morsprunorum*, and *persicae* (Gašić et al., 2012). The pathovar-specific primers based on *rhs* family gene sequences were also used to simultaneously identify the *Xanthomonas* species complex associated with tomato bacterial spot, including *X. vesicatoria*, *X. perforans*, and *X. gardneri* (Park et al., 2009; Araujo et al., 2012). To facilitate routine identification of *Erwinia* species, a PCR method based on species-specific sequences of the housekeeping genes *recA* and *gpd* was developed to differentiate *E. amylovora*, *E. pyrifoliae*, *E. billingiae*, *E. persicina*, *E. rhapontici*, and *E. tasmaniensis*. Moreover, differentiation using species-specific primers could be done via either conventional PCR (cPCR) or qPCR (Gehring and Geider, 2012). Moreover, multiplex qPCR with multiple fluorescent reporter dyes were developed to facilitate the simultaneous detection of different pathogenic species of genus *Pseudomonas* (Haan E.G.d and Bovenkamp G.W.v.d, 2009) and three important rice pathogens, *Xanthomonas oryzae* pv. *oryzae*, *X. oryzae* pv. *oryzicola*, and *Burkholderia glumae* (Luqi et al., 2014). A combination of specific primers and multiplex-PCR could identify *Pseudomonas syringae* pv. *morsprunorum*, *P. s.* pv. *lachrymans*, and *P. s.* pv. *syringae* from different host plants, different cultivars of sweet cherry, and oil pumpkin grown in different locations (Ilicic et al., 2016). However, a significant reduction in sensitivity was observed for pathogens in the presence of plant extract (Mcmanus and Jones, 1995). In recent years, loop-mediated isothermal amplification (LAMP) is developed for pathogen detection and disease diagnosis (Yang et al., 2025).

## Nanotechnology-based diagnostics

Immunological and molecular techniques have advanced but have some issues related to rapidity, signal strength and instrumentation (**Table 1**). Nanoparticles are different from their bulk counterparts when reduced to nanosize (1-100 nm). In nanosize, they possess certain properties suitable for their development as diagnostic probes (Sharon et al., 2010). Nanofabrication techniques had been used in creating artificial plant parts such as stomata and xylem vessel which are then used to detect pathogens [e.g., *Aspergillus niger* (Etefagh et al., 2013), *Xanthomonas campestris* pv. *vesicatoria* (Yao et al., 2009), and *Xylella fastidiosa* (Meng et al., 2005)] and monitor the infection process and behavior of pathogens inside host plants (Meng et al., 2005). A conjugation with a secondary antibody improves the sensitivity when detecting pathogenic bacteria causing bacterial spot on solanaceous plants (Yao et al., 2009). The integration of immunological and molecular diagnostics with nanotechnology systems offers an option where all detection steps can be accommodated on a portable miniaturized device for rapid and accurate detection of plant pathogens. However, the nanotechnology-based diagnostics for plant pathogens is still developing and the cost is relatively high.

## Emerging single-cell technology

Fluorescent hybridization was first introduced to detect plant pathogens at the single-cell level. Pathogens [e.g., *Pseudomonas cinnamomi* (Li AY. et al., 2014) and *Gamma proteobacteria* (Li et al., 2017)] can be specifically detected and visualized directly using fluorescent *in situ* hybridization (FISH) via a species-specific fluorescently labelled DNA probe. The advantage of FISH is that the plant or pathogen could retain integrity without damage and there is no need for subculturing. In contrast, it is difficult to localize the hyphae and reproductive structures of pathogens within plant tissues, especially in woody tissues (Li AY. et al., 2014). An additional microscopic method is solid-phase cytometry (SPC) which allows rapid detecting plant pathogen bacteria at the single cell level, without the need for a growth phase. After filtration of the sample, the retained microorganisms are fluorescently labeled on the membrane filter and automatically counted and identified by an epifluorescence microscope (D’Haese and Nelis, 2002). SPC shows equally accurate for the quantification of bacteria compared culture-based method (Vanhee et al., 2009). It has considerable advantages compared to the culture-based method, including its low detection limit (4 cells/m^3^), rapid (within 24 h), and the straightforward microscopic identification (Vanhee et al., 2009) (**Table 1**). Moreover, SPC in conjunction with fluorescent viability staining is powerful to detect viable but non-culturable microbes (Cools et al., 2005). In parallel, flow cytometric analysis was developed. When combined with fluorescent labels (e.g., propidium iodide), flow cytometry could detect plant pathogens in crude seed extracts without further extraction (Tebaldi et al., 2010). However, the abovementioned methods are very device-dependent as high-resolution electron microscopies are indispensable to accurately determine the labeled single cells (Li et al., 2017). Moreover, the inherent disadvantage of these methods is the difficulty to find proper labels and determine the proper labelling conditions which is time-consuming and technique-demanding. Therefore, recent research has focused on finding alternative label-free approaches for super-sensitive pathogen identification at the single-cell level.

Comprehensive biochemical information can be provided by noninvasive optical spectroscopy techniques, e.g., infrared, hyperspectral imaging and Raman spectroscopy (Kubryk et al., 2015; Wang et al., 2016) (**Table 1**). With very low background noise of aqueous samples, Raman spectroscopy is especially well-suited for biological applications (Wang et al., 2016). A single-cell Raman spectrum of bacterium represents a sum of Raman spectra of all cell components. It provides comprehensive information about the cell (e.g., nucleic acids, proteins, carbohydrates, and lipids), and could enable the distinction of various strains at the unicellular level with appropriate chemometrical methods based on comprehensive reference databases (Kloss et al., 2013; Kloß et al., 2015; Kubryk et al., 2015). It has been applied to detect bacteria in plants by identifying different spectra that are unique to each bacterium, much like fingerprint analysis (Jarvis and Goodacre, 2008; Gan et al., 2017).

**Figure 2:**
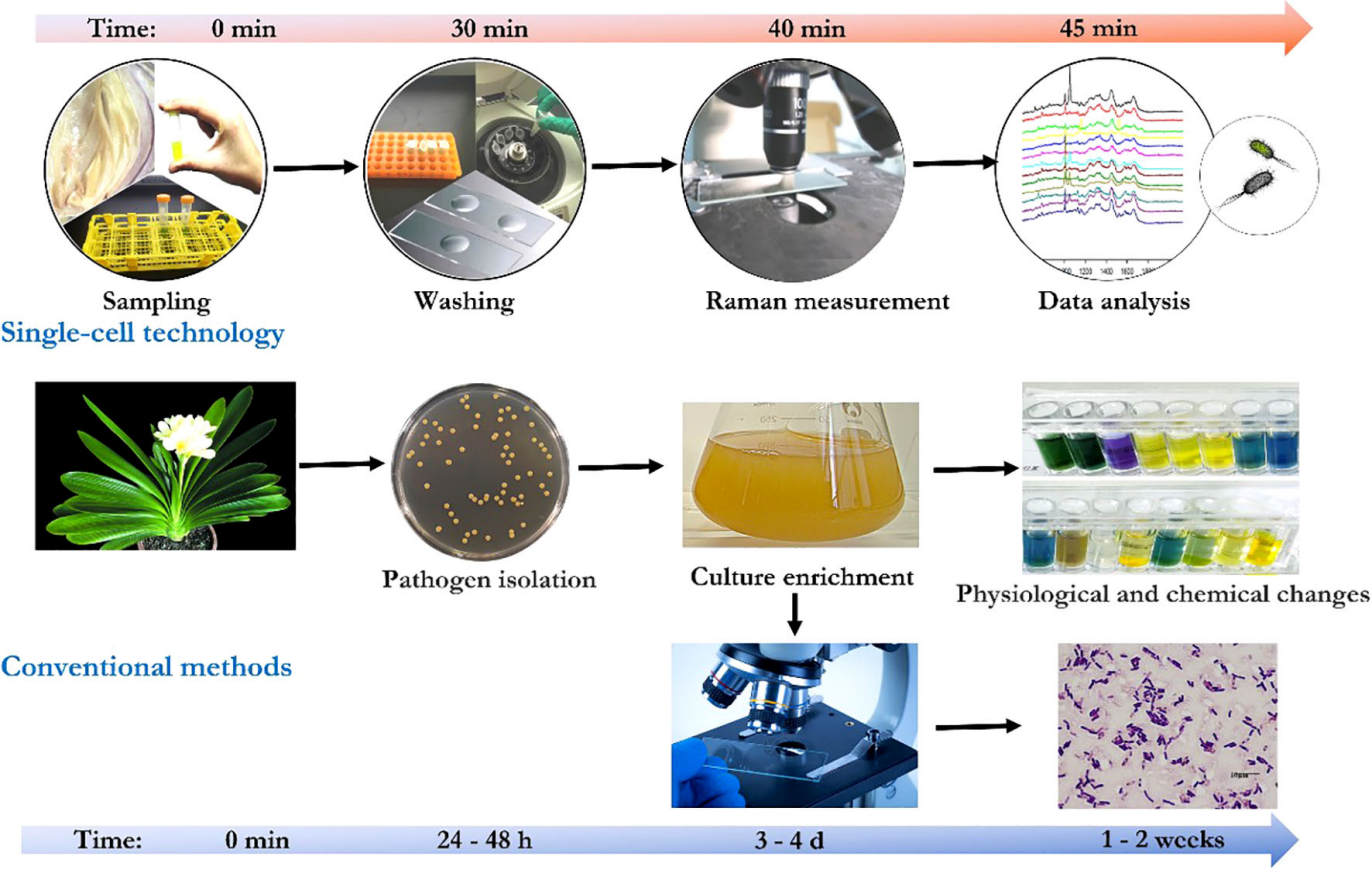
Comparison of conventional methods and the single-cell technologies for plant pathogen detection.

In particular, surface-enhanced Raman scattering spectroscopy (SERS) is able to enhance the signal by 11 orders of magnitude, which is sufficient for single-molecule detection (Blackie et al., 2009). SERS signal enhancement enables the detection of low concentrations of pathogenic bacteria in plant samples. Employing standardized protocol involving optimized parameters, such as mixing procedure of bacterial samples, concentrated colloidal suspension, and statistic analysis [e.g., principal component analysis (Guicheteau et al., 2008; Gan et al., 2017)], reproducible SERS spectra could be generated and numerous of plant pathogens have be identified from bacterial mixtures (Cam et al., 2010; Kang et al., 2010). Moreover, multiplex detection of agriculturally important plant pathogens (i.e., *Botrytis cinerea*, *Pseudomonas syringae*, and *Fusarium oxysporum*) was demonstrated by using SERS (Lau et al., 2016). Furthermore, micro-Raman spectroscopy-based bioassay could detect plant pathogens at single-bacterium level in plant tissue lesions without pre-enrichment (Gan et al., 2017a) which makes in-site identification of pathogens from real plants possible. Species-specific detection of either a single plant pathogen (Yüksel et al., 2015) or multiple pathogens (Lau et al., 2016) from infected plant materials was achieved via label-free SERS or the multiplex detection approach. In general, compared with conventional methods, with proper sample preparation, the single-cell technologies enable a highly sensitive, pre-enrichment-free, and real-time detection of plant pathogen at a single-cell level (**Figure 2**).

## Next-generation technologies for crop pathogen detection

Although morphology-based diagnostics (e.g., morphology-, antibody-, nucleic-acid-based methods) and emerging single-cell technology (e.g., fluorescent hybridization, solid-phase cytometry, optical spectroscopy techniques) have, in many ways, met the growing need for *in vitro* diagnostic tools for pathogens, the advance of single-cell sequencing and synthetic biology promise a revolution toward the development of new diagnostic tests for a fraction of the cost and time (Gan et al., 2017; Marcolungo et al., 2022). The development and successful application of single-cell sequencing has greatly expanded our knowledge of the diversity and phylogeny of microorganisms. It provides an alternative to culturing organisms as a prerequisite for genomic sequencing and allows discrimination between subspecies when present either individually or in combination. Pathogen detection and typing could be achieved within approximately ten minutes of sequencing owing to the use of an internal control (Marcolungo et al., 2022). However, there are still challenges facing the field, such as an efficient method to obtain single cells. To address the challenge, microfluidic platforms have been developed that enable the isolation, enrichment, and biochemical or genetic analysis of individual cells with high spatiotemporal resolution (Li et al., 2019). The combination of microfluidics and single-cell sequencing has been applied for the whole-transcriptome sequencing of animal embryonic cells (Streets et al., 2014), the diagnosis of cancers and immune system diseases (Keller and Pantel, 2019). However, its application in plant pathogen detection is just emerging.

Synthetic biology was recognized early on as an opportunity to engineer organisms that could serve as whole-cell biosensors (Attaluri and Dharavath, 2023). As synthetic biology has matured, increasing gene circuit complexity has allowed for greater sensitivity and reporter tunability of the synthetic biosensors (Cubero et al., 2024). Bacterial viruses harbor natural specificity to a wide range of bacterial pathogens and can be explored for diagnostic applications of pathogens. The correct bacterial surface epitope or pathogen-derived peptides serves as a conditional input that regulates the output of signals generated by luciferase or other reporter genes of the engineered synthetic phage (Pardee et al., 2014). Low-cost microfluidics coupled with a pathogen capture technology could further improve the sensitivity of the phage-based detection (Kang et al., 2014). Although the application of single-cell sequencing and synthetic biology in plant pathogen detection is just emerging, new frontiers in the rational design principles of these technologies have been ushered with a direct impact on applied and foundational studies relating to pathogen biology and diagnostics (Mukherjee and Schroeder, 2016).

## Field trials and commercialization

On-site diagnostic is a challenge for tangible application of plant quarantine. Progress is being made to address the challenge through transforming these diagnostic technologies into “outside-the-lab” application. For example, to identify pathogens in plant tissues (Llop et al., 2000) and soil (Puławska and Sobiczewski, 2005), a single-tube nested PCR was developed. It allowed reliable detection of pathogens with a number of as few as 100, and was unaffected by the presence of plant-tissue or soil-derived PCR inhibitors (Kositcharoenkul et al., 2011). More and more pathogens could be detected from real plant samples (Mirik and Aysan, 2009), even at the early stage of infection (Taylor et al., 2001) for different pathogens with similar symptoms (Umesha et al., 2015). PCR-based kits, such as Probelia™, for the detection of plant pathogens (e.g., *Erwinia carotovora* subsp. *atroseptica* in potatoes) have been evaluated at five laboratories in four countries. The kit was based on DNA-specific PCR amplification followed by detection of amplicons by hybridization to a peroxidase-labelled DNA probe in a microplate (Frechon et al., 1998). To increase the reproducibility, optimized protocol for DNA extraction from plants, specific primers, and procedure were used for pathogen detection in contaminated plants (Abd-Elsalam et al., 2011). Immunological assays have also been commercialized for the detection of a wide variety of pathogens due to that these methods are reliable and do not require highly trained personnel. The LAMP assay successfully detect American plum line pattern virus with crude flowering cherry extract (Garg et al., 2022). Nowadays, researchers achieved precise dual detection: One-tube reverse transcription-recombinase aided amplification (RT-RAA) combined with lateral flow strip (LFS) assay for RNA and DNA target genes from pepper mild mottle virus and Colletotrichum species in crude plant samples (Cao et al., 2025).

**Figure 3:**
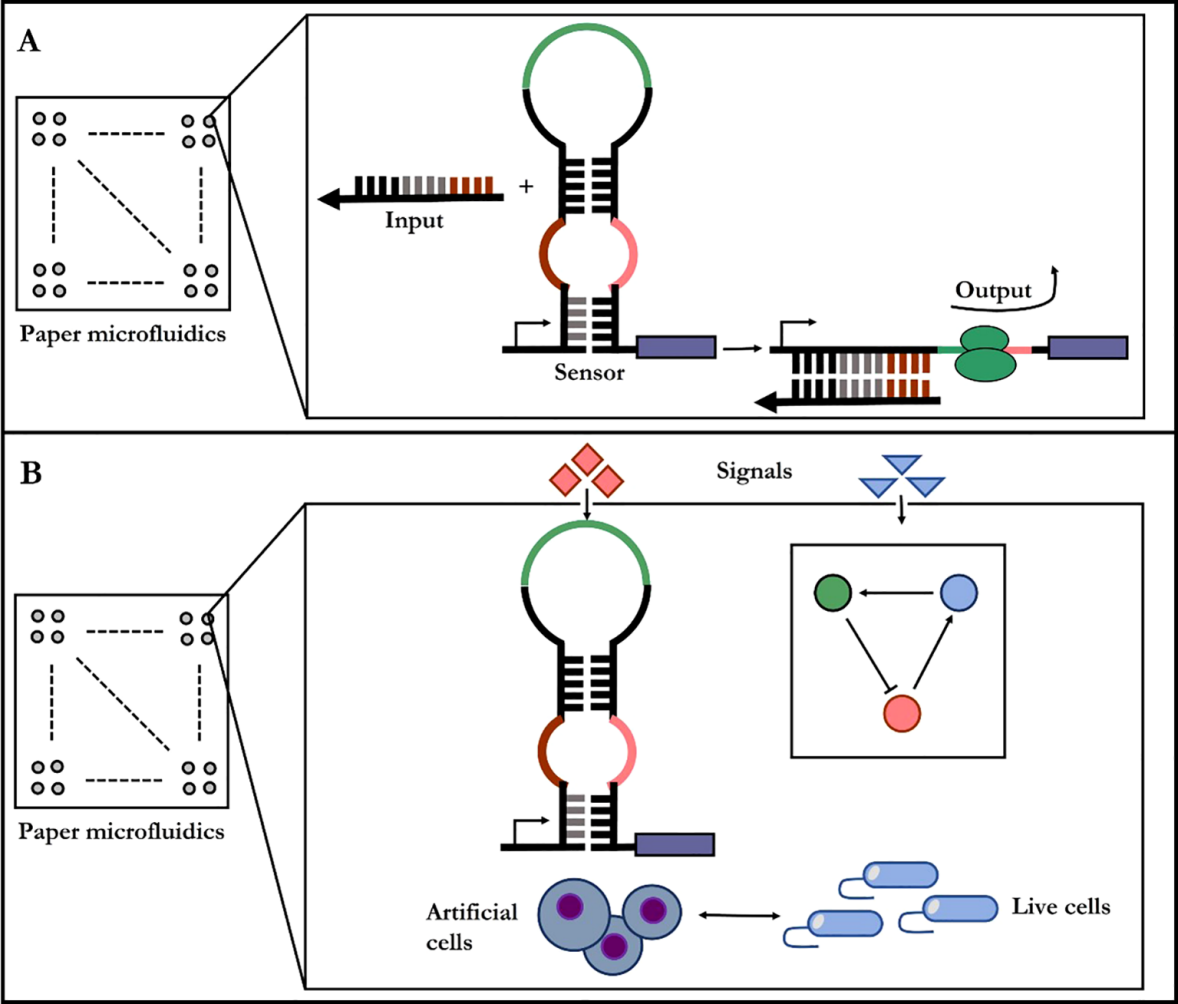
Paper-based diagnostic synthetic-biology designs. **(A)** The gene circuit becomes active when rehydrated with the test sample, containing target RNAs or small molecule. **(B)** Diagnostic gene networks and cell-free transcription/translation system are assembled into paper or other porous materials.

Synthetic phage can reproduce rapidly in fermenters (Schofield et al., 2012). Therefore, phage-based pathogen are now commercially approved products for microbial detection in industrial settings, and clinical applications have been demonstrated as well (Lu et al., 2013). An emerging trend for in-site diagnostic lies in *in vitro* synthetic biology where cellular context is completely removed and synthetic gene circuits are put on paper (**Figure 3**) (Pardee et al., 2014). The synthetic diagnostic systems are freeze-dried onto porous substrates to create poised genetic regulatory networks that are stable for long-term storage at room temperature and are activated by rehydration. The paper-based reactions could be merged with custom, low-cost electronics for quantification and automation of diagnostic reactions in the field. At the macroscopic scale, pathogen detection methods and plant disease forecasting in crop systems have been improved by using nanosensors which can be linked to a Global Positioning System for real-time monitoring of disease, soil conditions, and crop health (Abd-Elsalam, 2013). Despite these proof-of-concept demonstrations, several challenges remain for the practical implementation of these diagnostics, including meeting the detection thresholds required for field use.

## Concluding remarks and future perspectives

Detection of crop pathogens at the single-cell level is the latest goal of diagnostic technology at the present. Conventional diagnostics, such antibody- based techniques and PCR-based methods, are important and sensitive application methods. However, proper implementation of these techniques poses challenges, ranging from time-consuming bacteria enrichment to the significant cost of the technology. The most important is that these methods could not detect plant pathogenic bacteria at a single cell level.

We are witnessing a leapfrog development of pathogen detection from culture-based strategies to *in-situ* diagnostic when investigators begin to design FISH, SPC, and label-based optical spectroscopy experiments. These methods allow the detection of viable but non-culturable microbes in the field. However, the sensitivity and resolution needed for the experiments should be considered. The advent of microfluidic technologies, coupled with rapid advances in fluorescence-based molecular imaging, had spurred a revolution in biological analysis at the level of single cells (Khan, 2014). But these methods are label-dependent and technique-demanding.

The development of a micro-Raman spectroscopy-based bioassay enabled fast, label-free and non-invasive discrimination of plant pathogens, and accurate culture-free single-bacterium detection in plant tissue lesions with an identification ratio comparable to those of genetic molecular approaches (Gan et al., 2017). It signifies a trend towards the development of methods for *in situ*, real-time, and single-bacterium detection which offers exciting new opportunities in entry-exit quarantine, customs inspection, food-processing, medical diagnosis, and biological weapons inspections, crucial to national security. However, Raman spectroscopy requires capital funding for equipment. A systemic, standardized, and comprehensive spectroscopic database of cultured bacteria is always a precondition for measurements. The cultivation should include the respective environmental conditions and enough biological replicates to establish a classification model assessing the possibility to distinguish between taxa. Next generation sequencing also requires having a reference dataset of vouchered specimens for use in general diagnostics of unknown. Therefore, the capacity of Raman spectroscopy to detect unknown plant pathogens remains challenge.

As single-cell sequencing joins ranks with other diagnostics, its combination with microfluidic enables *in situ*, real-time, and single-cell detection of unknown microbes. On the other hand, synthetic biology provides low-cost, rapidly deployable diagnostics. The engineering approaches have the potential to significantly advance the translation of promising technologies into pragmatic tools suitable for real-world applications. The paper-based synthetic biosensor and portable hybrid devices incorporating synthetic sensors or single-cell sequencing fit the need for low-cost, practical, and simple diagnostic tools for use outside of the laboratory. It is also intriguing to imagine *in vitro* synthetic biology embedded into diagnostic wearables for crops, allowing for both in-site and real-time sensing. Success will continue to be found at the interfaces between disciplines.
